# Chronic Granulomatous Disease and Myelodysplastic Syndrome in a Patient with a Novel Mutation in *CYBB*

**DOI:** 10.3390/genes12101476

**Published:** 2021-09-23

**Authors:** Bárbara C. S. Reis, Daniela P. Cunha, Ana Paula S. Bueno, Flavia A. A. Carvalho, Juliana Dutra, Fabiana V. Mello, Maria Cecília Menks Ribeiro, Cristiane B. Milito, Elaine Sobral da Costa, Zilton Vasconcelos

**Affiliations:** 1 Instituto Fernandes Figueira (IFF/Fiocruz), Rio de Janeiro 22250-020, Brazil; barbaracsreis@gmail.com (B.C.S.R.); danielapradocunha@gmail.com (D.P.C.); flaviaanisio@yahoo.com.br (F.A.A.C.); jutdutra@gmail.com (J.D.); 2 Fundação Oswaldo Cruz, Rio de Janeiro 22250-020, Brazil; 3 Instituto de Puericultura e Pediatria Martagão Gesteira (IPPMG), Universidade Federal do Rio de Janeiro (UFRJ), Rio de Janeiro 21941-610, Brazil; apbueno65@gmail.com (A.P.S.B.); fabivmello@gmail.com (F.V.M.); cecilia.menks@gmail.com (M.C.M.R.); elainesc@medicina.ufrj.br (E.S.d.C.); 4 Faculdade de Medicina, Universidade Federal do Rio de Janeiro (UFRJ), Rio de Janeiro 21941-901, Brazil; crismilito@gmail.com

**Keywords:** chronic granulomatous disease, pediatric myelodysplastic syndrome, rare diseases

## Abstract

Chronic Granulomatous Disease (CGD) is an inborn error of immunity characterized by impaired phagocyte function, recurrent fungal and bacterial infections and granuloma formation in multiple organs. Pediatric myelodysplastic Syndrome (MDS) is a rare hematological stem cell disease that leads to an ineffective hematopoiesis with variable risk of evolution to acute leukemias. Both disorders are rare and have distinct pathophysiologic mechanisms, with no known association. A 7-month-old boy presenting with recurrent infections and anemia at age 2 months underwent immunological, hematological and genetic investigation that culminated in the diagnosis of both CGD and MDS. Next generation sequencing was performed and identified a silent variant predicted as of Uncertain Significance, located in the splicing site at the end of exon 5 in *CYBB*. *CYBB* variants account for at least two thirds of CGD cases, but no previous descriptions of this variant were found in ClinVar or The Human Gene Mutation Database (HGMD) databases. We were able to demonstrate an exon 5 skipping on the proband’s cDNA, which strongly suggests the disruption of the NADPH oxidase complex, abrogating the formation of reactive oxygen species from neutrophils. Moreover, erythroid cell lineage could be also affected by NADPH oxidase complex damages. Further investigation is needed to evaluate the potential effect of *CYBB* gene alterations in hematopoiesis, as well as in MDS and CGD association.

## 1. Introduction

Chronic Granulomatous Disease (CGD) and Myelodysplastic Syndrome (MDS) are both rare disorders with no known pathophysiologic link so far. CGD is an inborn error of immunity (IEI) caused by a defect in any of the five subunits of the NADPH oxidase enzyme complex (gp91phox, p22phox, p47phox, p67phox, p40phox), responsible for the phagocyte respiratory burst [1]. The disease’s prevalence is estimated in 1:120–250,000 [2] and in two thirds of patients it displays an X-linked pattern of inheritance, as a consequence of *CYBB* gene variants, which encodes the gp91phox protein. CGD patients are at increased risk of serious infections by bacteria and fungi, in addition to inflammatory complications. Clinical manifestations vary from skin infections to granulomas in internal organs and autoimmune diseases [3]. Treatment options include antibiotic and antifungal prophylaxis, interferon-gamma (IFN-γ), hematopoietic stem cell transplantation (HSCT) and gene therapy.

MDS is responsible for less than 5% of all hematological neoplasms of childhood [4]. Monosomy 7 due to a somatic loss is the commonest cytogenetic abnormality associated with childhood MDS. HSCT is the only curative treatment available and it is the therapy of choice in these cases and other complex cytogenetic abnormalities [5].

Here, we report a male patient that spontaneously developed both rare diseases in the first months of his life, describing the genetic evaluation and the strategies used for his diagnosis. To our knowledge there are no reports of other individuals presenting both diseases spontaneously; instead, all reported cases developed MDS with chromosome 7 monosomy only after gene therapy for CGD [6,7,8]. There are also many reports associating gene therapy with other haematological complications (e.g., lymphoma). Thus, it is the first description of MDS with chromosome 7 monosomy and CGD association in an untreated child.

## 2. Materials and Methods

### 2.1. Study Design

Case report of clinical features and next generation sequencing of a patient that presented with recurrent infections and anemia at age 2 months. The patient’s mother and sister were also evaluated in order to confirm the findings.

### 2.2. Clinical Evaluation

At age 2 months, the patient started presenting with anemia and monthly bacterial/fungal infections (candidiasis, pneumonia and urinary tract infections). By the age of 6 months, he was transferred to a tertiary hospital’s ICU presenting with sepsis and anemia. At this point, he was evaluated by hematology, immunology and genetics specialists, being diagnosed with CGD and MDS with chromosome 7 monosomy.

### 2.3. Genomic DNA Preparation

Peripheral blood samples from the patient and his mother and sister were collected in EDTA tubes. The patient’s genomic DNA was extracted from peripheral blood leucocytes using PureLink^®^ Genomic DNA Mini Kit Thermo Fisher (Thermo Fisher Scientific, Waltham, MA, USA), according to the manufacturer’s protocol. DNA concentration in samples were determined by fluorometry using Invitrogen Qubit^®^ 4 Fluorometer (Thermo Fisher Scientific, Waltham, MA, USA). To assess DNA purity, Spectrophotometer NanoDrop^®^ 2000 (Thermo Fisher Scientific, Waltham, MA, USA). was used to evaluate the ratio of the absorbance at 260/280 nm (average of 1.90 for all samples) and at 260/230 nm (average of 1.91 for all samples). DNA samples were stored at 4 °C prior to use.

### 2.4. Library Preparation and Clinical Exome Sequencing

DNA libraries from both parents and children were prepared with 50 ng of DNA using Illumina TruSeq^®^ Exome according to the manufacturer’s recommendations (Illumina, San Diego, CA, USA). Sequencing was performed using NextSeq^®^ 500/550 system (Illumina, San Diego, CA, USA) using a multiplex system with 16 samples per run, with NextSeq^®^ kit. During library preparation, DNA fragments of 400 bp long on average were evaluated by Bioanalyzer by Agilent.

### 2.5. Bioinformatics Analysis

Sequencing data was processed and analysed using BaseSpace from Illumina and the genetic variants calls were performed by comparison with a reference sequence of hg19 from the University of California Santa Cruz (UCSC) Genome Browser. To analyse the patient’s variants found, the first strategy considered was filtering data using a virtual gene panel from the International Union of Immunological Societies Expert Committee (IUIS) early 2020 Human Inborn Errors of Immunity update [9]. During variant interpretation we considered allele frequency using Exome Aggregation Consortium database (ExAC), 1000 Genomes Project database, gnomAD and ABraOM, an online archive of Brazilian mutations (http://abraom.ib.usp.br/, accessed 8 June 2021). Fourteen predictors were considered for pathogenicity: CADD, BayesDel_addAF, DANN, DEOGEN2, EIGEN, FATHMM-MKL, LIST-S2, M-CAP, MVP, MutationAssessor, MutationTaster, SIFT and PrimateAI. Clinical significance of variants was evaluated with ClinVar, Polymorphism database (dbSNP), Human Gene Mutation Database (HGMD), Human Splicing Finder v.HSF3.0 [10] and Alamut splicing predictors (SSF, MES, NNSplice, GeneSplicer and EX-SKIP (Sophia Genetics, Saint Sulpice, Switzerland).

### 2.6. RNA Extraction and cDNA Synthesis

Total RNA was extracted with TRIzol^TM^ reagent (Life Technologies, Carlsbad, CA, USA) following the manufacturer’s recommendations. The RNA was quantified and analyzed for purity later in a spectrophotometer (Nanodrop Technologies, Wilmington, DE, USA).

With the RNA already extracted, we proceeded with the synthesis of the cDNA strands, which was carried out using the commercial kit Super Script III Invitrogen™ ^®^ (Thermo Fisher Scientific, Waltham, MA, USA) using random primers to prepare the first strand of cDNA, and following the instructions recommended by the manufacturer.

### 2.7. PCR and Sanger Sequencing

To evaluate the mRNA 5′ broken donor splicing site, the cDNA from the patient and a control were submitted to a polymerase chain reaction (PCR) using primers targeting the exon 4 (Forward Primer: 5′-GTTCGAAGACAACTGGACAGGA-3′) and exon 6 (Reverse Primer: 5′-GACCTCCGGATGGTTTTGGT-3′). The PCR products were visualized on 1.5% agarose gel after electrophoresis.

To evaluate mRNA decay, the cDNA from the patient and healthy control were submitted to a quantitative polymerase chain reaction (PCR) using primers targeting the *CYBB* exon 2 Forward Primer: 5′-CATTCTGGTTTGGCTGGGGT-3′ and exon 3 Reverse Primer: 5′-TTTCGACAGACTGGCAAGAGA-3′ and the GADPH Forward Primer: 5′-AGTGATGGCATGGACTGTGGTCAT-3′ and Reverse Primer: 5′-CAACAGCCTCAAGATCATCAGCAA-3′ with PowerUp™ SYBR^®^ Green Master Mix (Thermo Fisher Scientific, Waltham, MA, USA). The assay was performed in the Applied Biosystems 7500 Real-Time PCR System (Thermo Fisher Scientific, Waltham, MA, USA).

To confirm the suspected pathogenicity of the variant found, validation was done with the polymerase chain reaction (PCR) combined with bidirectional Sanger sequencing. Primers targeting the mutation site were designed for PCR-amplification (ThermoFisher Cycler, Waltham, MA, USA) and sequencing (Forward Primer: 5′-GGAATGGTGTGTGAATGCCC-3′ and Reverse Primer: 5′-CAGACTTGTCCCTCTGATGGT-3′). Primers and PCR products were purified using PureLink^®^ Invitrogen™ (Thermo Fisher Scientific, Waltham, MA, USA) and on automated sequencer ABI 3730 Genetic analyzer (Thermo Fisher Scientific, Waltham, MA, USA). The results were interpreted by software BioEdit.

### 2.8. DHR Assay

The neutrophil oxidative activity by flow cytometry aims to quantify the production of oxidative radicals by peripheral blood neutrophils after ex vivo stimulation with phorbol myristate acetate (PMA). This quantification takes place through a probe called dihydrorhodamine 123 (DHR) which generates rhodamine 123 as a product of the reaction. The negative result confirmed the diagnosis of CGD. In this patient, DHR was analyzed with Attune flow cytometry (Thermo Fisher Scientific, Waltham, MA, USA). Healthy donor samples stimulated in the presence of DHR were used as control.

### 2.9. FISH

Fluorescence in situ hybridization test (FISH) was used to detect chromosomal abnormalities such as microdeletions, complex rearrangements or absence of chromosomes. In this work, probes D7Z1 and D8Z2 were used, which detect chromosomes 7 and 8 respectively, thus rendering possible the identification of the patient’s chromosome 7 monosomy.

### 2.10. Flow Cytometry

For immunophenotypic analysis, the bone marrow (BM) sample was stained with an 8-colour combination, following the standard operating procedures proposed by the EuroFlow consortium [11]. Immediately after staining, cells were acquired in a FACSCantoII flow cytometer using the FACSDiva software (BD). INFINICYT™ software (Cytognos, Salamanca, Spain) was used for data analysis.

### 2.11. Ethics

The study was approved by the Ethics Committee in Research of Instituto Nacional de Saúde da Mulher, da Criança e do Adolescente Fernandes Figueira (IFF/Fiocruz). Written informed consents were obtained after explanation of the nature of the study. All procedures were conducted in accordance with the Declaration of Helsinki.

## 3. Results

**Figure 1:**
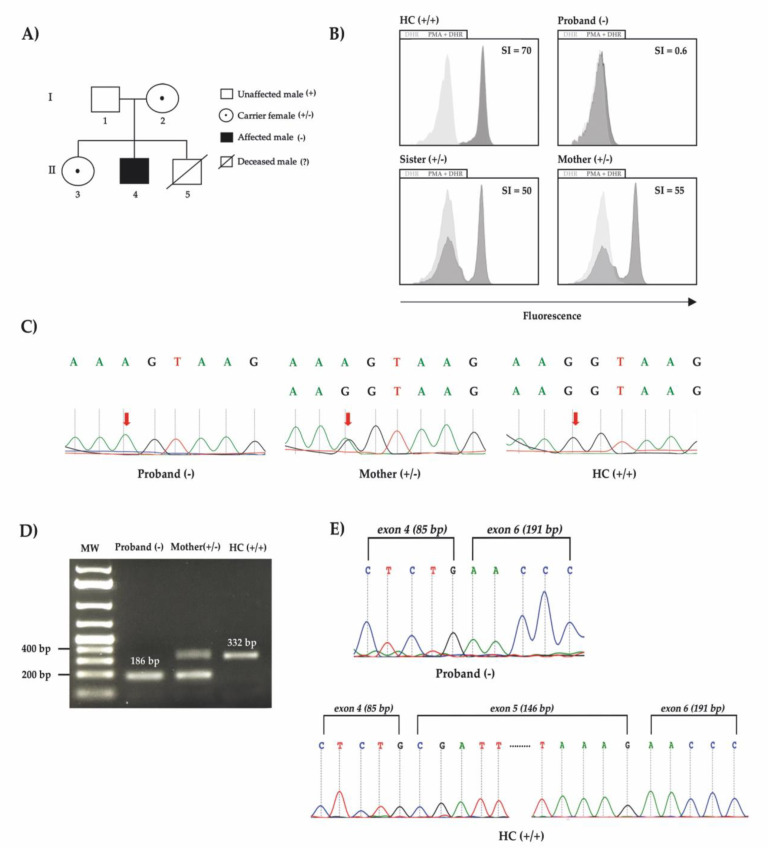
(**A**) The proband’s family pedigree of affected patients displaying two generations. White circles with central dots represent carriers, the proband is represented as a black square. (**B**) Flow cytometry was used to assess fluorescence intensity increase after neutrophils stimulation with PMA in presence of DHR123 (grey histogram) and control with DHR only (faint histogram). In the healthy control, the curve shifts to the right, showing a neutrophil’s response. The proband’s curves before and after stimulation overlap, showing that there was no phagocyte respiratory burst. The curves of the proband’s mother and sister partially shift, revealing their carrier state, seeing as, because of their heterozygous variant, part of their neutrophils responds but the rest does not. SI = stimulation index. (**C**) Electropherogram from DNA showing homozygous variant on the proband, heterozygous variant on the proband’s mother, a mutation carrier, and homozygous wild type allele in a healthy control. (**D**) cDNA PCR products confirming a small fragment from the proband compatible with exon 5 skipping in comparison to a healthy control (HC) on agarose gel. Healthy control (HC), proband’s mother and sister were positive, while the proband was negative, suggesting intronic retention. MW: molecular weight. (**E**) Electropherogram from cDNA showing exon 5 skipping on the proband and continuous exon 4 to 6 in a healthy control (HC).

**Figure 2:**
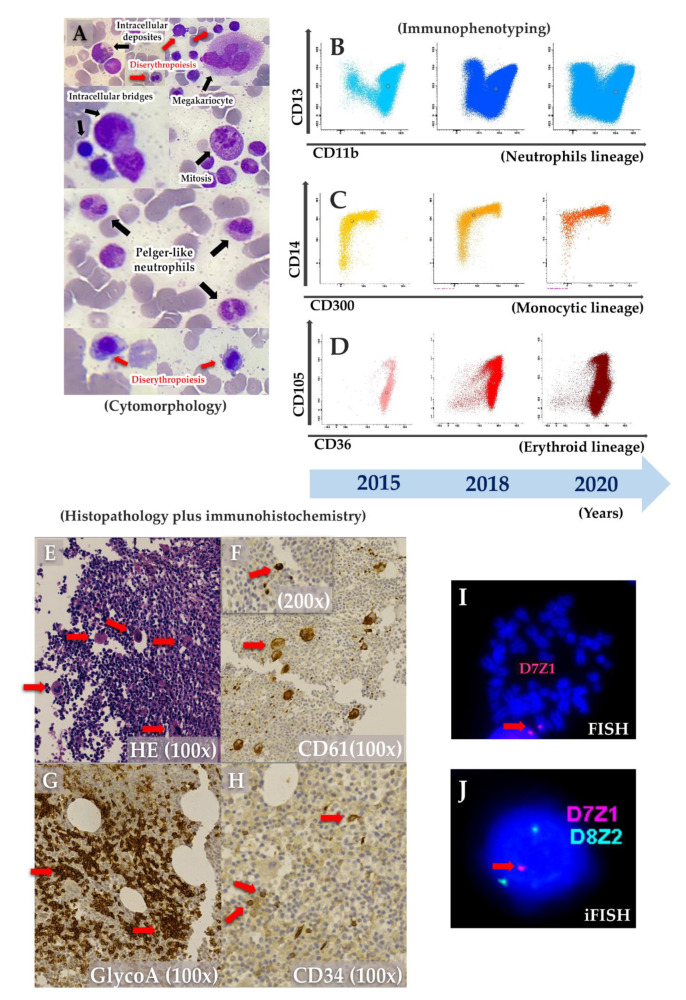
Bone Marrow (BM) aspirate was characterized with (**A**) cytomorphology stained with MayGrunwald-Giemsa (MGG) that revealed myelodysplastic characteristics (dyserythropoiesis, dysmegakaryopoiesis, intercellular bridges, Pelguer-huet-like neutrophils and mitosis figure); (**B**–**D**) immunophenotyping sequentially performed a long of time that showed maintained maturative blockage in neutrophils (**B**), monocytes (**C**) and erythroid lineages (**D**). BM histopathology (**E**) stained with hematoxylin-eosin (HE) showed normal cellularity with atypical megakaryocytes. CD61 staining (**F**) showed small clusters of megakaryocytes and micromegakaryocytes. Glycophorin A (**G**) marked large and irregular groups of erythroblasts and CD34 (**H**) stained progenitor cells (1–2%). FISH (**I**) and interphase iFISH (**J**) showed chromosome 7 monosomy.

The patient was first admitted to the hospital when he was 2 months old with anemia and urinary tract infection. From there on, he presented with anemia and monthly bacterial/fungal infections (candidiasis, pneumonia and urinary tract infections) receiving red blood cells transfusions on two occasions (4 and 5 months old). His brother’s death at 10 months old after pneumonia (Figure 1A), along with the patient’s diagnosis of fungal sepsis, motivated immunologic investigation. By the age of 6 months, he was transferred to a tertiary hospital’s ICU with pulmonary sepsis, without spleen or liver enlargement and with confirmed anemia (hemoglobin—8.6g/dL; WBC 8.1 × 10^9^/L; platelets count—186 × 10^9^/L; neutrophils count 3.6 × 10^9^/L; LDH-737 U/L), and being treated with broad spectrum antibiotics and hemotransfusion. Immunoglobulins and lymphocytes immunophenotyping were normal. Bone marrow (BM) aspirate revealed dyserythropoiesis, dysmegakaryopoiesis, Pelger-huet-like neutrophils and intercellular bridges (Figure 2A). Further BM immunophenotyping showed maturative blockage in neutrophils (between III/IV stages), monocytes (between stages II/III stages) and erythroid lineages (between I-II/III stages) (Table 1 and Figure 2B–D). On the monocytic maturation, there was an aberrant expression of CD105 in the entire maturation (Table 1). On histopathology, BM was normocellular with atypical megakaryocytes (Figure 2E). In immunohistochemistry, CD61 staining showed small clusters of megakaryocytes, and micromegakaryocytes and glycophorin A staining showed large and irregular erythroblasts grouped (Figure 2F–H). Cytogenetic analysis suggested a chromosome 7 monosomy, which was confirmed by FISH and iFISH (25% of all analysed nuclei) (Figure 2I–J). Taken all together, the patient was diagnosed with primary MDS with chromosome 7 monosomy and refractory cytopenia without blasts excess, according to World Health Organization (WHO) classification.

Next generation sequencing (NGS) of the whole exome was then performed, ruling out the presence of mutations in genes related to primary MDS, as well as other hereditary diseases that affect myelopoiesis (e.g., Fanconi anemia, Blackfan-Diamond anemia etc) (Table S1). In contrast, NGS revealed a novel synonymous variant c.483G>A/p.(Lys161=) in *CYBB*, the main gene involved in X-linked CGD (XL-CGD), that was predicted as of Uncertain Significance by The American College of Medical Genetics and Genomics (ACMG). It was observed that the variant was located in a potential 5′ donor splice site, which raised suspicion that, despite it being a silent variant, it might still affect gene function and protein formation. In silico analysis was performed using Human Splicing Finder (HSF v3.0) and Visual Alamut softwares and all predictors suggested a 5′ donor splicing site with a high score (HSF = 97, SSF = 94, MES = 10, NNSplice = 1 and GeneSplicer = 5). All predictors also showed results indicating a broken donor site alteration, most probably affecting splicing, with the following ratios: HSF = −10.4%, SSF = −12.9%, MES = −28.5%, NNSplice = −0.9% and GeneSplicer = −88.2%. Additionally, Alamut EX-SKIP predictor indicated that mutated alleles (ESS/ESE ratio = 0.62) have a higher chance of exon skipping than wild type alleles (ESS/ESE ratio = 0.57).

To proceed with immunologic investigation, we performed a dihydrorhodamine (DHR) test, the gold standard for diagnosing CGD. The test uses flow cytometry to measure fluorescence before and after neutrophils incubated with dihydrorhodamine 123 (DHR) are stimulated with phorbol myristate acetate (PMA). Phagocyte’s respiratory burst depends on NADPH-oxidase proper functioning and produces hydrogen peroxide that oxidizes DHR into rhodamine 123, which emits fluorescence, as seen in Figure 1C with a healthy control. Moreover, the test is able to distinguish XL-CGD, autosomal recessive CGD (AR-CGD), and X-linked carrier status (in females). The proband’s DHR was negative, suggesting CGD diagnosis. DHR also demonstrated a characteristic dual fluorescence profile, typical of an XL-CGD carrier, on the patient’s mother and sister (Figure 1B).

To validate the variant, we performed Sanger sequencing with DNA samples from a healthy control, the proband’s mother and the proband. We were able to demonstrate the proband’s homozygous variant, the mother’s electropherogram showing heterozygosity, confirming a healthy carrier, and a healthy control with homozygous wild type allele (Figure 1C).

In order to demonstrate the exon skipping that would justify the patient’s phenotype, a polymerase Chain Reaction (PCR) of cDNA was done using samples from the patient and a healthy control. To compose the reaction, a specific pair of primers were used targeting the exon 4 (forward primer) and exon 6 (reverse primer) to amplify the surrounding splice region in the case of exon 5 exclusion together with the proband RNA processing. As shown in Figure 1D, the amplicon from the proband cDNA displayed a smaller size of 186 bp compatible with exon 5 skipping in comparison to the mother’s with 332 bp and 186 bp, and the healthy control with a single fragment with 332 bp. We further checked the RNA decay by qPCR targeting the exon 2 to exon 3 and the proband showed a Log_2_ Fold Change of 2.9 in comparison to the healthy control. This result could explain the mother’s smaller fragment dominance after amplification (Figura 1D). Finally, both amplicon’s Sanger sequencing demonstrated the exon 4 sequence followed by exon 6 in the proband sample, contrasting with the healthy control product that showed a continuous exon 4 to 6 electropherogram data (Figure 1E).

After diagnosis and the start of prophylactic antibiotics and antifungals, our patient had exquisite clinical improvement, remaining well and with no new admissions for the first four years of his life. Nevertheless, at age 5, the patient’s clinical status started to deteriorate as he presented, in the months that followed, with failure to thrive, pneumonia, lymphadenopathy and rib osteomyelitis, being admitted to the hospital and treated with broad spectrum antibiotics and antifungals on several occasions, as well as being evaluated for typical and atypical mycobacteriosis. At this point, we started efforts to search for a compatible bone marrow donor for a stem cell transplant, the only widely available curative treatment for CGD so far.

## 4. Discussion

Curiously, our patient presented with CGD, an IEI, and primary MDS with chromosome 7 monosomy. Both diseases are rare in children, share no known pathophysiologic link and presented in the patient’s first few months of life. Despite this, MDS often causes aberrancies in neutrophil differentiation that can generate neutropenia, neutrophils dysmorphisms and severe infections [4]. The impaired phagocyte function in neutrophils, characteristic of CGD, could potentially also occur in MDS, but is not routinely studied in such patients.

Different genotypes in CGD may present with clinical manifestations in distinct frequencies. Berg et al. [12] described the largest CGD cohort so far, with 429 European patients. Of those, 82% were male, which reflects the main form of the disease’s inheritance, which is X-linked, involving *CYBB* variants. AR-CGD appears to display a milder phenotype, which may explain the significant difference in mean onset age, higher on AR-CGD than on XL-CGD (Chronic Granulomatous Disease: The European Experience). As described for XL-CGD, our case had an early onset with severe infections such as pneumonia, sepsis and urinary tract infections. However, after antibiotic and antifungal prophylaxis started, our patient remained well until 5 years-old, when his clinical status deteriorated, with failure to thrive, lymphadenopathy, pneumonia and osteomielitis. Despite the fact that our patient presented with some of the most frequent clinical manifestations of CGD largely described from others (e.g., pneumonia, lymphadenopathia, sepsis and osteomyelitis) [12,13,14], he presented also with a chronic anemia that was due to primary MDS with chromosome 7 monosomy, in absence of mutations in genes related to primary MDS, as well as other hereditary diseases that affect myelopoiesis, investigated by whole exome sequencing.

In the present case, MDS affected not only phagocytic lineages (neutrophils and monocytes), but also erythroid and megakaryocytic lineages. Both the presence of monosomy 7 and aberrancies in non phagocytic cells discarded the hypothesis of CGD mimicking MDS in BM, seeing as CGD alterations are restricted to phagocytes. Another possible hypothesis was that one genetic aberration could lead to the two conditions. In consonance with this hypothesis, monosomy 7 could not be the principal alteration responsible for the two conditions, since it was present in only 25% of hematopoietic cells, while the baby had the whole hematopoiesis altered. Furthermore, monosomy 7 disappeared three years after, even though dysplastic alterations remained for two more years. That emphasized the need for a deep genetic investigation.

By whole exome sequencing, we were able to detect a silent variant in *CYBB*. This type of mutation is frequently ignored, since the substitution leads to the same amino acid and therefore theoretically won’t cause disease. However, because of the variant’s location, at a potential donor splicing site, we further investigated using in silico and molecular strategies. Five in silico predictors suggested a broken 5′ donor splice site alteration with high scores ratios and one exon skipping predictor indicated a higher chance of exon skipping on the mutant allele compared with the wild type sequence. Using the cDNA from the family members, we were able to demonstrate an exon skipping mechanism in a specific region surrounding the splicing site only in the proband sample, indicating the probable exon removal during mRNA processing, which highly suggests an explanation for the patient’s phenotype. This exon removal generates one premature stop codon with a truncated protein at the 115 position. Despite the fact that it was a synonymous variant, intronic removal or exon retention is an essential step during the pre-mRNA processing for correct protein encoding. Any disruption on this highly controlled cellular process could impair protein function, as shown here by functional assays performed with proband neutrophils. This is not the first time that a splice-site variant was found to cause a patient to present with clinical and laboratory evidence of CGD. Rawat et al. reported 15.8% patients diagnosed with CGD that presented with splice-site variants in *CYBB* [14], showing that splice variants are even less frequent in this context. This patient prevalence is also true of Clinvar genetic variant frequency, where, in a total of 105 pathogenic or likely pathogenic variants described in the *CYBB* gene, 28 (26.6%) were missense, 26 (24.7%) were nonsense, 15 (14.2%) were splice-site (like ours) and only 1 (0.95%) was an indel related variant.

Interestingly, Stein et al. [6] reported in 2010 two adult male patients with X-linked CGD treated with gene therapy in Frankfurt. During follow-up, both subjects presented with peripheral blood abnormalities that later led to the diagnosis of MDS with chromosome 7 monosomy, the exact same cytogenetic defect as our patient. Stein et al.’s findings suggested that, in at least one progenitor cell (probably myeloid), the MDS1-EVI1 gene locus was transcriptionally activated by gammaretroviral insertion leading to chromosome 7 monosomy and MDS [6]. Their data showed that forced over-expression of EVI1 in human cells disrupts normal centrosome duplication with consequent genomic instability, chromosome 7 monosomy and clonal progression toward MDS [15]. The MDS1-EVI1 gene locus is also a common target for tumorigenesis produced by a wild-type retrovirus or retrovirus vector-induced insertional mutagenesis [7]. Those findings suggest that there might still be an unknown correlation between CGD and MDS, perhaps through insertional activation of certain genes by a wild or vector-induced retrovirus.

## 5. Conclusions

Our data clearly demonstrated an exon 5 skipping mechanism occurring on the *CYBB* gene of a XL-CGD patient with neutrophil NADPH oxidase complex functional loss and MDS displaying a chromosome 7 monosomy due to a somatic loss and affecting phagocytic, erythroid and megakaryocytic lineages. From our knowledge, this is a unique case of MDS and CGD spontaneous association, and more studies are needed to evaluate the potential effect of *CYBB* gene alterations in hematopoiesis.

## Figures and Tables

**Table 1:** Bone marrow immunophenotype results.

Bone Marrow Aspirate (Cell Subtypes)	Percentage (%)	Differentiation (%|Stage)	Specific Subtypes (%)
Neutrophils	65.8	0.2|I	
		3.3|II	
		29.4|III	
		32.9|IV	CD10 = 15.40
Eosinophils	3.8		
Monocytes	5.0	0.7|I 2.6|II 1.6|III	CD34 = 0.08 CD105 = 5
Erythroblasts	3.0	0.5|I-II 2.6|III	
Basophils	0.1		
Dendritic cells	0.3		
T cells	3.1		
B cells	7.8		
NK ^1^ cells	0.2		
Lymphoid CD34 ^2^ cells	1.2		
Myeloid CD34 ^2^ cells	1.0		CD33 = 0.30 CD7 = 0.10 CD64 = 0.01

^1^ Natural killer; ^2^ Cluster Differentiation.

## Data Availability

The data presented in this study are available on request from the corresponding author. The data are not publicly available due to IRB requests.

## Supplementary Materials

The following are available online at https://www.mdpi.com/article/10.3390/genes12101476/s1, Table S1: List of 67 genes related to MDS available at Human Phenotype Ontology (diseases included: Fanconi anemia, Primary MDS/AML, Thrombocytopenia 2, Thrombocytopenia 5, Familial aplastic anemia, Shwachman-Diamond syndrome, Diamond Blackfan anemia, Congenital neutropenia, Familial platelet disorder, Myeloid neoplasms with germline predisposition).

## References

[1] Chiriaco M., Salfa I., Di Matteo G., Rossi P., Finocchi A. (2016). Chronic Granulomatous Disease: Clinical, Molecular, and Therapeutic Aspects. Pediatr. Allergy Immunol..

[2] De Oliveira-Junior E.B., Zurro N.B., Prando C., Cabral-Marques O., Pereira P.V.S., Schimke L.-F., Klaver S., Buzolin M., Blancas-Galicia L., Santos-Argumedo L. (2015). Clinical and Genotypic Spectrum of Chronic Granulomatous Disease in 71 Latin American Patients: First Report from the LASID Registry. Pediatr. Blood Cancer.

[3] Arnold D.E., Heimall J.R. (2017). A Review of Chronic Granulomatous Disease. Adv. Ther..

[4] Hasle H. (2016). Myelodysplastic and Myeloproliferative Disorders of Childhood. Hematol. Am. Soc. Hematol. Educ. Program.

[5] Chatterjee T., Choudhry V.P. (2013). Childhood Myelodysplastic Syndrome. Indian J. Pediatr..

[6] Stein S., Ott M.G., Schultze-Strasser S., Jauch A., Burwinkel B., Kinner A., Schmidt M., Krämer A., Schwäble J., Glimm H. (2010). Genomic Instability and Myelodysplasia with Monosomy 7 Consequent to EVI1 Activation after Gene Therapy for Chronic Granulomatous Disease. Nat. Med..

[7] Ott M.G., Schmidt M., Schwarzwaelder K., Stein S., Siler U., Koehl U., Glimm H., Kühlcke K., Schilz A., Kunkel H. (2006). Correction of X-Linked Chronic Granulomatous Disease by Gene Therapy, Augmented by Insertional Activation of MDS1-EVI1, PRDM16 or SETBP1. Nat. Med..

[8] Siler U., Paruzynski A., Holtgreve-Grez H., Kuzmenko E., Koehl U., Renner E.D., Alhan C., van de Loosdrecht A.A., Schwäble J., Pfluger T. (2015). Successful Combination of Sequential Gene Therapy and Rescue Allo-HSCT in Two Children with X-CGD—Importance of Timing. Curr. Gene Ther..

[9] Bousfiha A., Jeddane L., Picard C., Al-Herz W., Ailal F., Chatila T., Cunningham-Rundles C., Etzioni A., Franco J.L., Holland S.M. (2020). Human Inborn Errors of Immunity: 2019 Update of the IUIS Phenotypical Classification. J. Clin. Immunol..

[10] Desmet F.O., Hamroun D., Lalande M., Collod-Béroud G., Claustres M., Béroud C. (2009). Human Splicing Finder: An online bioinformatics tool to predict splicing signals. Nucleic Acids Res..

[11] Van Lochem E.G., van der Velden V.H., Wind H.K., te Marvelde J.G., Westerdaal N.A., van Dongen J.J. (2004). Immunophenotypic differentiation patterns of normal hematopoiesis in human bone marrow: Reference patterns for age-related changes and disease-induced shifts. Cytom. Part B Clin. Cytom..

[12] Van den Berg J.M., van Koppen E., Ahlin A., Belohradsky B.H., Bernatowska E., Corbeel L., Español T., Fischer A., Kurenko-Deptuch M., Mouy R. (2009). Chronic granulomatous disease: The European experience. PLoS ONE.

[13] Winkelstein J.A., Marino M.C., Johnston R.B., Boyle J., Curnutte J., Gallin J.I., Malech H.L., Holland S.M., Ochs H., Quie P. (2000). Chronic granulomatous disease. Report on a national registry of 368 patients. Medicine.

[14] Rawat A., Vignesh P., Sudhakar M., Sharma M., Suri D., Jindal A., Gupta A., Shandilya J.K., Loganathan S.K., Kaur G. (2021). Clinical, Immunological, and Molecular Profile of Chronic Granulomatous Disease: A Multi-Centric Study of 236 Patients From India. Front. Immunol..

[15] Dunbar C.E., Larochelle A. (2010). Gene therapy activates EVI1, destabilizes chromosomes. Nat. Med..

