# Supplementary material for: Chronic Granulomatous Disease and Myelodysplastic Syndrome in a Patient with a Novel Mutation in CYBB
Source: Genes (Basel). 2021 Sep 23;12(10):1476. doi: 10.3390/genes12101476 (PMC8535487; doi:10.3390/genes12101476)
Supplement: Supplementary file 1 [file genes-12-01476-s001.zip › genes-1254620-supplementary.pdf]

Supplementary material

Table S1. List of 67 genes related to MDS available at Human Phenotype Ontology (diseases included: Fanconi anemia, Primary MDS/AML, Thrombocytopenia 2, Thrombocytopenia 5, Familial aplastic anemia, Shwachman-Diamond syndrome, Diamond Blackfan anemia, Congenital neutropenia, Familial platelet disorder, Myeloid neoplasms with germline predisposition)

ADA2	FANCL	RPS10
ANKRD26	FANCM	RPS15A
ATG2B	FLT3	RPS17
BRCA1	GATA1	RPS19
BRCA2	GATA2	RPS24
BRIP1	GFI1	RPS26
CTNNA1	GSKIP	RPS27
DDX41	IRP1	RPS28
DNAJC21	KIT	RPS29
EFL1	NF1	RPS7
ELANE	NRAS	RUNX1
ERCC4	PALB2	SBDS
ETV6	RAD51	SLX4
FANCA	RAD51C	SRP54
FANCB	RFWD3	SRP72
FANCC	RPL11	TCIRG1
FANCD2	RPL15	TET2
FANCE	RPL18	TSR2
FANCF	RPL26	UBE2T
FANCG	RPL27	WT1
FANCI	RPL35	XRCC2
KMT2A	RPL35A	
MAD2L2	RPL5	
